# Research on Dynamic Compressive Performance and Failure Mechanism Analysis of Concrete after High Temperature and Rapid Cooling

**DOI:** 10.3390/ma15134642

**Published:** 2022-07-01

**Authors:** Shuai Peng, Zhenpeng Yu, Qi Zhao, Xiaoqing Du, Xinghua Xie, Bo Chen, Yongping Zhang

**Affiliations:** 1 School of Mechanics and Engineering Science, Shanghai University, Shanghai 200444, China; pengshuai@shu.edu.cn (S.P.); yuzhenpeng@shu.edu.cn (Z.Y.); zq18575557212@163.com (Q.Z.); dxq@shu.edu.cn (X.D.); 2 Nanjing Hydraulic Research Institute, Nanjing 210029, China; iamxiexh@163.com; 3 Hangzhou Dingchuan Information Technology, Hangzhou 310020, China; booc8009@163.com

**Keywords:** rapid cooling after high temperature, concrete, compression, dynamic performance, failure mechanism

## Abstract

To investigate the dynamic compressive properties of concrete after high temperature and rapid cooling, an experimental study was carried out by considering five temperatures and four strain rates. The coupling effect of high temperature and strain rate on concrete damage morphology and mechanical parameters was comparatively analyzed. The main conclusions are as follows: the compressive damage morphology of concrete is affected by strain rate development trends of significant variability under different temperature conditions. As the strain rate increases, the compressive stress and elastic modulus of concrete are gradually increased. As the temperature increases, the increase in compressive stress is gradually reduced by the strain rate. For the temperatures of 20 °C and 800 °C, the increase in compressive stress by the strain rate is 38.69% and 7.78%, respectively. Meanwhile, SEM and CT scanning technology were applied to examine the mechanism of the effect of high temperature and strain rate on the mechanical properties of concrete from the microscopic perspective, and the corresponding constitutive model was proposed.

## 1. Introduction

As one of the commonest building materials, concrete is widely used in structural engineering, bridge engineering and water conservancy engineering. Concrete structures are not only subjected to static forces, but may also be subjected to dynamic effects such as earthquakes, shocks and explosions. Concrete has an obvious rate dependence, and its mechanical properties will change significantly under the effect of strain rate [1,2,3]. Moreover, concrete structures are often impacted by secondary disasters after dynamic loads, such as fires, floods and mudslides, among which fire is a major type. After a fire disaster, the mechanical properties of concrete will deteriorate, and the structural bearing capacity will be seriously affected. Therefore, comprehensive analysis of the mechanical properties of concrete in terms of high-temperature degradation under the influence of dynamic loads has great significance for evaluating the safety performance of concrete structures [4,5].

The research on the dynamic mechanical properties of concrete is generally carried out from two angles besides experiment and theory. Abrams et al. [6] found that the strength parameters and deformation parameters of concrete materials were significantly affected by the strain rate through compression tests. Watstein [7] and Malvar et al. [8] studied the dynamic mechanical properties of concrete under uniaxial compression and uniaxial tension, respectively, and the results showed that the strain rate of concrete under tension was significantly higher than that under compression. Li et al. [9] carried out compression and tension experimental research on the mechanical properties of concrete to reveal its failure mechanism and established a concrete dynamic constitutive model based on the elastoplastic damage theory. Lin et al. [10] and Song et al. [11] examined the dynamic mechanical properties of concrete by considering multi axial effects and proposed the corresponding failure criteria. The results showed that the confining pressure had a significant effect on the dynamic properties of concrete. Chen et al. [12] used Hopkinson compression bars to carry out experimental research on the dynamic mechanical properties of concrete under tension and compression, and proposed a dynamic tension and compression model by taking into account the damage evolution.

Research on the dynamic mechanical properties of concrete has derived fruitful results. However, concrete structures sometimes may suffer extreme fire conditions during normal service. The internal composition of concrete may change greatly under the action of high temperature, resulting in deterioration of mechanical properties. Therefore, research on the mechanical properties of concrete by considering the coupling effect of high temperature and strain rate has important value to practical engineering [13,14,15]. Sakr and El-Hakim [16] investigated the effect of high temperature duration and high temperature influencing factors on the basic mechanical properties of ilmenite concrete and reported that ilmenite concrete had good resistance to high temperature. Li, M. et al. [17] conducted an experimental study on the basic mechanical properties of high strength concrete after high temperature and analyzed the effects of temperature, moisture content and specimen size on the mechanical properties of concrete. Naser and Uppala [18] applied machine learning methods to examine the post-high temperature mechanical properties of different types of concrete and steel and proposed a model for calculating the residual properties of these materials after fire. Bastami et al. [19] studied the test data of post-high temperature concrete mechanical properties under compression and tension and established a high temperature intrinsic structure model. Bastami and Aslani et al. [20] carried out an experimental study on the post-high temperature mechanical properties of ultra-high performance concrete and compared it with ordinary concrete and high performance concrete. The results showed that the deterioration rate of the compressive strength of ultra-high performance concrete after high temperature was relatively higher, while the deterioration rate of the tensile strength was relatively lower.

Regarding the static mechanical properties of concrete after high temperature, a main point of view in the current research is that the effect of high temperature can increase the plastic deformation capacity of concrete and deteriorate its mechanical properties. When the temperature is lower than 400 °C, as the temperature increases, the mechanical properties of concrete will not change obviously; when the temperature exceeds 400 °C, as the temperature increases, the deterioration of the mechanical properties of concrete will become significant [21,22]. The research on the dynamic performance of concrete after high temperature was mainly based on the investigation of the compressive or tensile mechanical properties of concrete under high strain rates after high temperature. The results showed that the strain rate effect of concrete was significantly affected by the high temperature effect (the strain rate effect is gradually weakened as the temperature increases), and a constitutive model based on the coupling effect of high temperature and strain rate was proposed [23]. The literature [24] conducted a study on the dynamic mechanical properties of concrete after high temperature using the rapid cooling method, and the results showed that there were obvious differences in the strain rate effect among different cooling methods. However, all the studies above were carried out from the perspective of high strain rates, and the cooling methods considered were generally natural cooling. In actual engineering practice, concrete structures may be subjected to wind, earthquake and impact, as well as other dynamic actions under low and medium strain rates. In case of a fire disaster, the fire can be usually controlled in a short time, and rapid water cooling is usually adopted as the control method. Therefore, the dynamic mechanical performance of concrete under the cooling mode needs to be studied more comprehensively.

In this paper, a hydraulic servo machine and an industrial camera were used to conduct dynamic compression tests on concrete after different high temperature and rapid cooling treatments by considering the dynamic effects of low and medium strain rates. The stress-strain curves were obtained from the tests to extract the compressive stress, elastic modulus and peak strain data, and to examine the coupling effect of high temper-ature and strain rate on the dynamic mechanical properties of concrete under compres-sion from qualitative and quantitative perspectives. Then, the SEM and CT scanning technology were used to reveal the mechanism of dynamic compressive action of concrete after high temperature and rapid cooling from a microscopic perspective. At the same time, the dynamic compressive constitutive model of concrete after high temperature was proposed based on the ZWT model.

## 2. Materials and Methods

### 2.1. Specimen Preparation

In this paper, the design strength of ordinary concrete is 40 MPa, and the raw materials used in the preparation of test specimens are mainly cement, water, fine aggregate and coarse aggregate. The cement is ordinary silicate cement P. O 42.5 grade; fine aggregate is natural river sand (fineness modulus: 2.3~3.0; average particle size: 0.35~0.5 mm); coarse aggregate is natural aggregate gravel (particle size: 4~16 mm). According to the “Specification for Mix Proportion Design of Ordinary Concrete (JGJ55-2011)”, the concrete mix proportion for C40 strength grade was determined in this paper as shown in Table 1.

The test specimens in this paper were poured according to the above mix proportion. Firstly, cement and fine aggregate were poured into the mixer in turn and mixed well, and then coarse aggregate was poured into the mixer and mixed well again. Thereafter, the weighed water was poured into the mixer slowly, mixed well and poured into the wooden mold. Then, the concrete mixture was fully vibrated and compacted by the pounding bar. After 24 h, the concrete mixture was demolded and placed into the standard curing room for curing. The temperature of the curing room was controlled at 20 ± 3 °C and the humidity was controlled at 95%. The specimens were taken out immediately after 28 days of curing for testing in this paper.

### 2.2. Test Methodology

**Figure 1:**
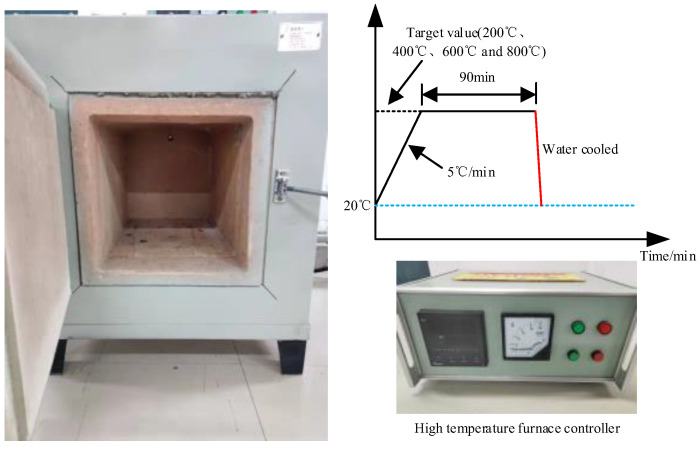
Concrete high temperature treatment technique and process.

The high temperature treatment process of concrete mainly includes three stages. The first stage is pretreatment. The test specimens were placed in a high temperature box for 2 h at 100 °C. The main consideration was to dry the moisture in the concrete to control the appearance of the burst of concrete during the heating process. The second stage is heating treatment. The heating method in this paper was determined by referencing the “Fire-resistance Tests—Elements of Building Construction (GB/T 9978-2008)”. The pre-treated specimens were placed in a high-temperature furnace, and the heating rate was set to 5 °C/min. After reaching the preset temperature, the specimens were stably placed in a high temperature furnace for 90 min. In this paper, five different temperature effects were considered, which are 20 °C, 200 °C, 400 °C, 600 °C and 800 °C, respectively. The third stage is the cooling stage. The high-temperature-treated test specimens were put in water for rapid cooling treatment first, and then dried and left to stand for 7 days. After the three stages of treatment above, the experiment was carried out immediately. The high-temperature treatment technique and process in this paper are shown in Figure 1.

Different strain rate intervals correspond to different dynamic response effects. In this paper, the compressive dynamic performance of concrete after high temperature was studied. Specifically, the dynamic action of seismic response magnitude was selected (the corresponding strain rate interval is 10^−3^/s~10^−2^/s), and accordingly, the test strain rates in this paper were set to be 10^−5^/s, 10^−4^/s, 10^−3^/s and 10^−2^/s, respectively, where 10^−5^/s refers to the static loading strain rate. The working condition was set as the static reference working condition [25]. The loading process can be divided into two steps. The first step is preloading: the load control method was used to load and unload the test specimen three times in order to control the influence of the gap between the loading surface of the equipment and the surface of the test specimen on the test results. The second step is formal loading: the displacement control loading method was used to load the test specimen according to the above-mentioned strain rates, and the loading stopped after the specimen was broken. In view of the conditions of the equipment used and the setting of the concrete specimen size in reference to the relevant literature, the specimen size was set as 70.7 mm × 70.7 mm × 70.7 mm.

In this paper, five high temperature conditions and four strain rate conditions were considered for each high temperature condition. Considering the randomness and discreteness of concrete materials, three specimens were prepared for each condition and the mean value was taken for the analysis. Therefore, the total number of test specimens is 60.

### 2.3. Test Equipment

**Figure 2:**
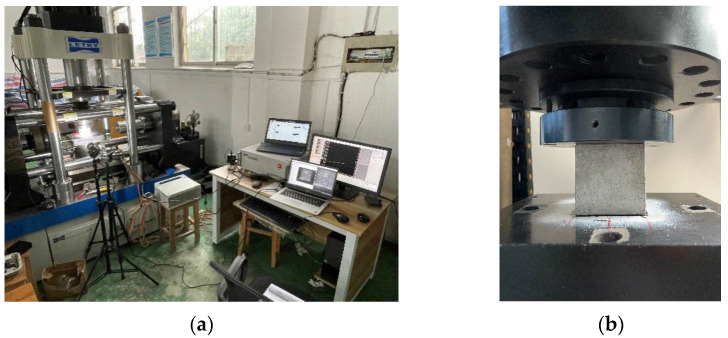
Test equipment and loading schematic diagram. (**a**) Loading equipment and industrial camera; (**b**) loading schematic diagram.

The hydraulic servo machine was used to conduct the uniaxial compressive dynamic performance test of concrete after high-temperature treatment. The equipment was assembled with a two-way loading end; the equipment of each loading side included a load sensor and displacement sensor. The test was completed using the axial loading end, the maximum load range of the axial device was 100 tons, the accuracy was ±0.1% of the maximum load range, the maximum displacement of the axial device was 100 mm and the accuracy was at most ±0.0005 mm. In order to acquire accurate strain data, strain gauges were arranged axially on the surface of the specimen to obtain the concrete deformation data during the loading process. Overall, the test equipment satisfied the accuracy requirements of load and deformation during concrete loading, as shown in Figure 2.

The temperature and loading strain rate have obvious effects on the compressive failure mode of concrete and also have certain effects on stress development during the stress process. In order to better study the coupling effect of temperature and strain rate on the macro mechanical properties of concrete, an industrial camera was used to record the concrete loading course to obtain the whole concrete damage evolution process under different loading conditions. The resolution of this camera is 4096 × 3000 pixels, and the maximum acquisition frequency is 180 HZ, which can satisfy the test requirements in this paper.

## 3. Test Results and Analysis

### 3.1. Static Working Conditions

**Figure 3:**
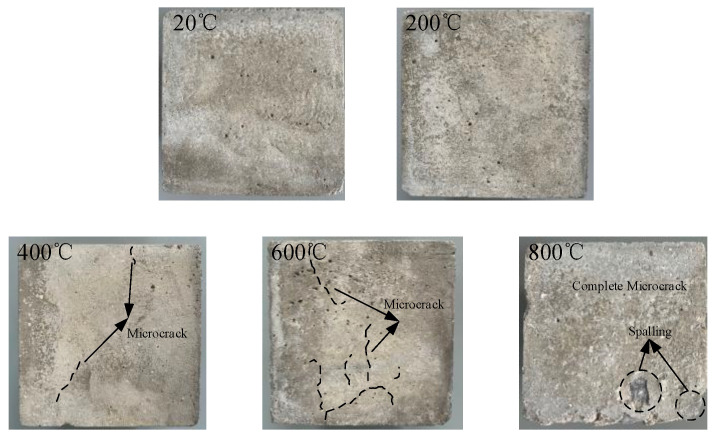
Concrete surface damage morphology after high temperature and rapid cooling.

**Figure 4:**
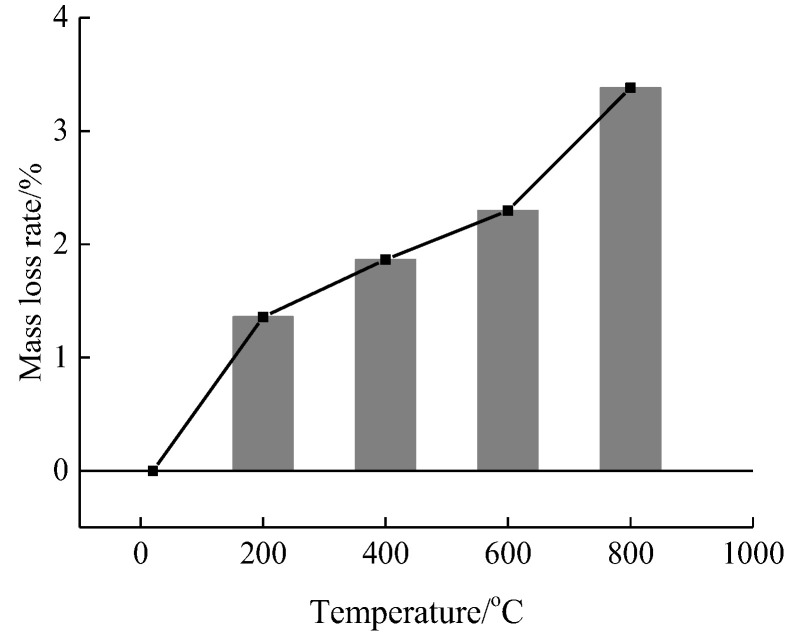
Temperature and concrete mass loss rate.

**Figure 5:**
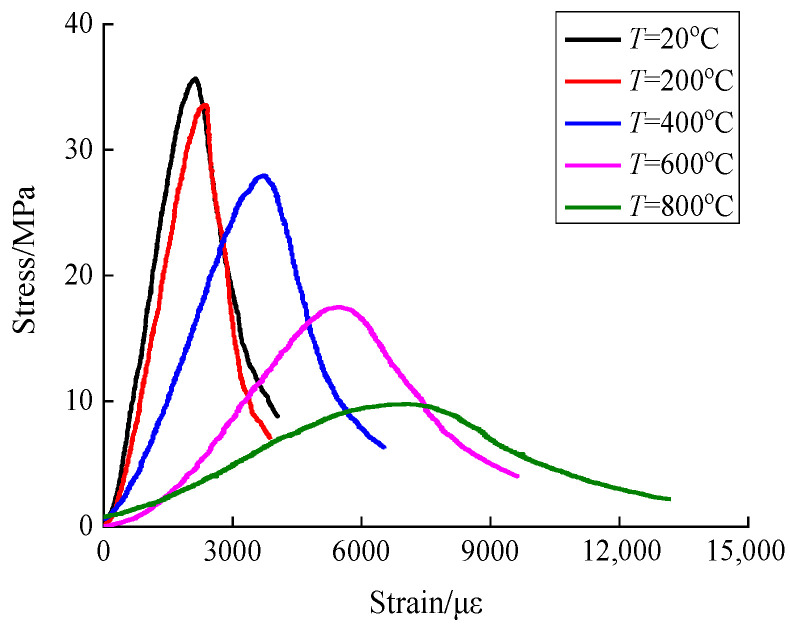
Stress-strain curve of concrete under static conditions.

The concrete surface damage morphology as well as the mass loss rate was obtained after different high temperature and rapid cooling concrete treatments, as shown in Figure 3 and Figure 4. The stress-strain curves of concrete after different high temperatures were examined from the perspective of static loading strain rate, and the static mechanical properties were analyzed, as shown in Figure 5.

According to Figure 3, when the temperature is 20 °C, the concrete surface presents a gray-brown color. When the temperature is raised to 200 °C, the color of the concrete surface does not change significantly after rapid cooling treatment. When the temperature is raised to 400 °C, the concrete surface presents a light yellow color after rapid cooling treatment, and there exist one or two micro cracks on the surface. When the temperature is raised to 600 °C, the yellow color of the concrete surface becomes more obvious after rapid cooling treatment, and there are more micro-cracks distributed on the surface. When the temperature is further raised to 800 °C, the concrete surface becomes gray-white after rapid cooling treatment, with distribution of crack networks on the surface accompanied by a small number of broken fragments and spalling of the edges. Compared with the morphology of concrete under natural cooling after the action of high temperature as reported in the literature [26,27], the cracks formed under rapid cooling are relatively more obvious. As the test specimen will be cooled down when it encounters water after high temperature, an obvious temperature gradient will be formed from the outside to the inside, and the resulting temperature stress will make the cracks formed on the inner surface different from that under natural cooling. The mass loss rate of the test specimens after rapid cooling treatment under different high-temperature conditions was then obtained and analyzed, as shown in Figure 4. According to Figure 4, the concrete mass loss rate is 1.39% when the temperature is 200 °C, 1.86% when the temperature is 400 °C, 2.30% when the temperature is 600 °C, and 3.38% when the temperature is 800 °C. With the rise of temperature, the mass loss rate is gradually increased. Compared with the mass loss rate under natural cooling as reported in the literature, the mass loss rate under rapid cooling is relatively higher, mainly due to the larger temperature gradient formed by rapid cooling, which makes the test specimens more prone to spalling and therefore mass loss.

In order to control the high temperature cracking or spalling of the test specimens during the temperature elevation process, all the specimens were dried in advance before the high temperature treatment to prevent high moisture content from causing cracking and spalling. Therefore, in this paper, no specimen burst or spalled during the temperature elevation process. High-temperature cracking and spalling is mainly due to the thermal stress and steam pressure caused by the action of inconsistent internal and external pressure. The phenomenon produced in this paper is the chemical reactions in the cement mortar and coarse aggregate under the action of high temperatures and their uneven deformation to produce damage and cracking. High temperatures can lead to cracking damage or bursting and spalling of test specimens, both having a significant effect on the deterioration of the mechanical properties of concrete.

The compressive stress-strain curves of concrete after rapid cooling at different high temperatures under static conditions are shown in Figure 5. The compressive stress-strain curves corresponding to different temperatures are divided into three phases, i.e., the elastic phase, the elastic-plastic phase and the decreasing phase. From the preliminary analysis of the stress-strain curve, with the rise of temperature, the concrete compressive stress is gradually reduced, especially at 400 °C, and the peak strain is gradually increased. High temperature makes the test specimen exhibit a more apparent plastic deformation capacity.

Then, the envelope area of the stress-strain curve was calculated to determine the effect of temperature on the strain energy density of concrete under stress. The strain energy density of concrete is 7575 KJ at room temperature, and 6346 KJ, 9374 KJ, 8524 KJ and 7423 KJ when the temperature is 200 °C, 400 °C, 600 °C and 800 °C, respectively. As the temperature increases, the concrete stress is gradually reduced and the strain value is gradually increased. According to the findings of this paper, it can be seen that the effect law of high temperature on the strain energy density of concrete under stress is relatively discrete, showing no clear pattern.

**Figure 6:**
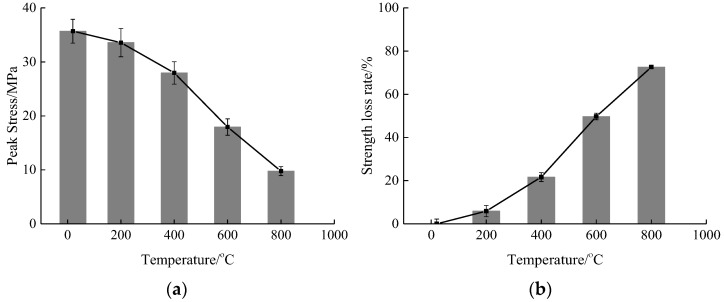
Relationship between temperature and compressive stress of concrete under static working conditions. (**a**) Compressive stress; (**b**) stress loss rate.

The compressive stress data of concrete was extracted from the stress-strain curves at different high temperature working conditions under the static strain rate, in order to analyze the effect of high temperature on the compressive stress, as shown in Figure 6.

According to Figure 6, the compressive stress value of concrete is 35.69 MPa at 20 °C, and 33.58 MPa, 27.96 MPa, 17.94 MPa and 9.77 MPa at the temperatures of 200 °C, 400 °C, 600 °C and 800 °C, respectively. Compared to the room temperature condition, the compressive stress is reduced by 5.91%, 21.66%, 49.73% and 72.63%, respectively. From the overall trend analysis, it can be seen that with the rise of temperature, the compressive stress is gradually reduced. When the temperature exceeds 400 °C, the decrease in compressive stress due to temperature is significantly increased. Compared with the decrease in compressive stress under natural cooling as reported in the literature [28,29], the decrease in compressive stress under rapid cooling is relatively higher due to the temperature effect. The main reason is that the concrete is affected more significantly by the temperature gradient under the high-temperature rapid cooling mode and the initial damage of concrete is relatively higher, so that its compressive stress is lower than that under natural cooling under the influence of temperature.

### 3.2. Damage Patterns

**Figure 7:**
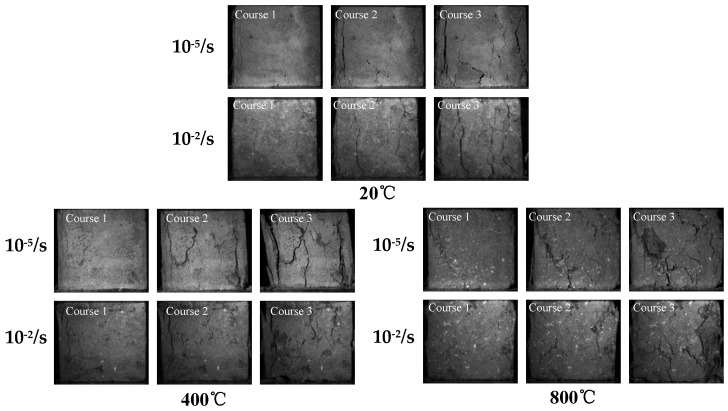
Damage process of concrete under the influence of different temperatures and strain rates.

**Figure 8:**
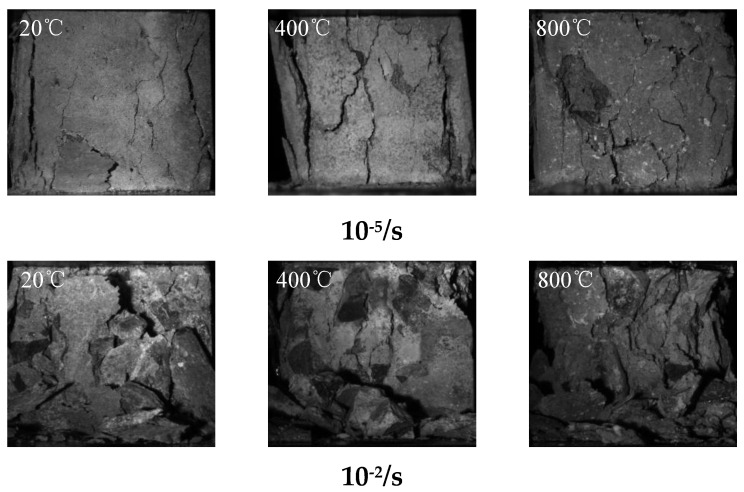
Final damage morphology of concrete under different temperatures and strain rates.

In this paper, an industrial camera was used to capture the compressive damage process of concrete under different temperatures and strain rates, so as to analyze the coupling effect of strain rate and temperature on the macroscopic mechanical properties of concrete. Unlike traditional damage morphology analysis, this technique can obtain the whole crack generation and damage evolution process. Considering the space limit of the paper, 20 °C, 400 °C and 800 °C were selected as the temperature conditions, and the strain rates 10^−5^/s and 10^−2^/s were selected as the static and dynamic conditions respectively, as shown in Figure 7 and Figure 8.

According to Figure 7, when the temperature is 20 °C and the strain rate is 10^−5^/s, the specimen first forms longitudinal cracks in the edge area, and as the load further develops, oblique discrete micro-cracks are formed in the middle area. At last, small fragments will peel off from the surface and wide longitudinal cracks are formed in the edge areas on both sides. When the temperature is 20 °C and the strain rate is 10^−2^/s, the concrete surface firstly forms longitudinal micro-cracks; with the development of load, longitudinal cracks gradually appear and penetrate the specimen, and finally, the concrete surface is distributed with a certain number of longitudinal cracks. When the temperature is 400 °C and the strain rate is 10^−5^/s, the concrete surface firstly forms longitudinal micro-cracks in the edge area. With the development of load, deep diagonal cracks and a small number of micro-cracks are uniformly distributed in the middle area of the test specimen, and at the same time, a longitudinal crack is generated on the edge of the other side. Finally, the cracks begin to gradually penetrate the specimen. When the temperature is 400 °C and the strain rate is 10^−2^/s, the concrete surface firstly forms longitudinal and transverse cracks at the edge area. With the development of load, discrete oblique cracks are formed in the middle area of the specimen, and finally, more uniformly-distributed cracks appear on the surface of the specimen but the degree of crack penetration is relatively low. When the temperature is 800 °C and the strain rate is 10^−5^/s, the concrete surface firstly generates diagonal cracks in the edge area, which develop toward the middle area, forming more micro-cracks near the diagonal crack area and other areas of the specimen. With the development of load, broken fragments will eventually peel off and the cracks will penetrate the specimen diagonally. When the temperature is 800 °C and the strain rate is 10^−5^/s, the initial concrete cracks are uniformly distributed in the middle area of the specimen. With the development of load, the cracks in the middle area begin to evolve gradually, and finally, discrete diagonal cracks are formed on the surface of the specimen accompanied by the appearance of spalling.

According to Figure 8, the static damage morphology of concrete is significantly different after different high temperatures. With the rise of temperature, the number of cracks formed in the specimen is gradually increased, and the distribution and development direction of cracks become more discrete. When the temperature is 20 °C, there are relatively fewer cracks, and the development direction is mainly longitudinal. When the temperature is 400 °C, the number of concrete cracks is increased, and the cracks mainly develop in the longitudinal and oblique directions. When the temperature is 800 °C, the number of cracks is the largest among all temperature conditions, and there are not only cracks developing along the longitudinal and oblique directions, but also a small number of lateral cracks, which are relatively slender. When the strain rate is 10^−2^/s and the temperature is 20 °C, the test specimen is damaged with a certain amount of coarse aggregate peeling off. When the temperature is 400 °C, the damage to coarse aggregate is significantly reduced, with existence of a small amount of coarse aggregate completely striped. When the temperature is 800 °C, the specimen is damaged with a large amount of coarse aggregate peeling off, but there is basically no coarse aggregate damage.

The failure mechanism above is attributed to the temperature gradient in the test specimen formed by the high temperature and rapid cooling action. The higher the temperature, the greater the concrete stress gradient, and the number and size of cracks formed on the surface and inside of the concrete specimen is relatively large. Under the effect of the static force, the stress path extends from the initial damage site in the process of concrete stress; there is a certain randomness and dispersion in the initial damage evolution of concrete after high-temperature rapid cooling, so that the damage pattern of concrete under pressure after high-temperature rapid cooling is definitely different from that in normal temperature conditions. Under the dynamic action, some cracks are formed in concrete because of the rapid cooling from the high temperature. The cracks are generally distributed in the interface between the mortar and coarse aggregate; the existence of cracks causes some changes in the stress path transmission of concrete, which is significantly different from that in normal temperature conditions. As a result, the failure of concrete under high strain rate conditions generally occurs at the interface between the mortar and coarse aggregate, accompanied by parts of the coarse aggregate peeling off. There is a certain similarity between the static and dynamic conditions.

### 3.3. Stress-Strain Curve

**Figure 9:**
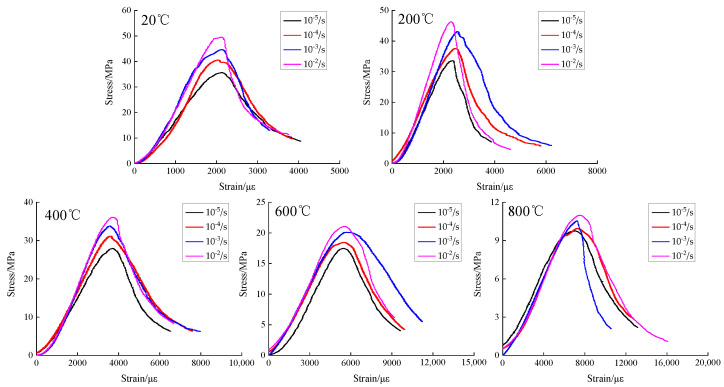
Compressive stress-strain curves of concrete under different temperatures and strain rates.

Considering five different rapid cooling effects after high temperature and four different strain rates, a material hydraulic servo machine was used to acquire the concrete load and deformation data under different working conditions. Then, the compressive stress-strain curves of concrete under different temperatures and strain rates were obtained, as shown in Figure 9.

According to Figure 9, the effect of temperature on the development pattern of compressive stress-strain curves of concrete with different strain rates is almost the same as that in static conditions; in all of them, the concrete compressive stress has a gradual decreasing trend, the peak strain gradually increases, and the concrete plastic deformation capacity increases, but there is no effect on the development trend of concrete compressive stress-strain curves. Under the same temperature but different strain rates, as the strain rate increases, the compressive stress is gradually increased. From the overall trend analysis, the increase of temperature makes the compressive stress of concrete have a gradually decreasing trend with the increase of strain rate, the peak strain by the strain rate is more discrete, and the strain rate has no effect on the development trend of the compressive stress-strain curve of concrete.

### 3.4. Peak Stress

**Figure 10:**
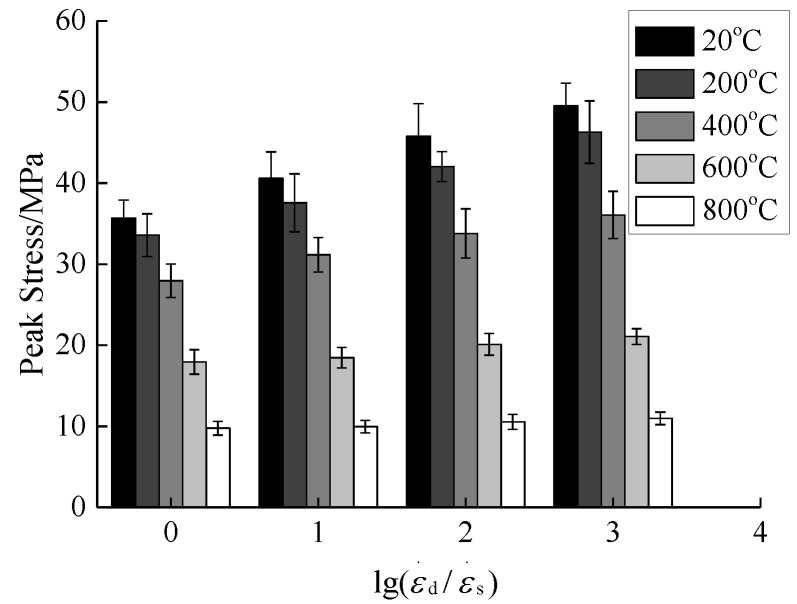
Relationship between strain rate and concrete compressive stress.

**Figure 11:**
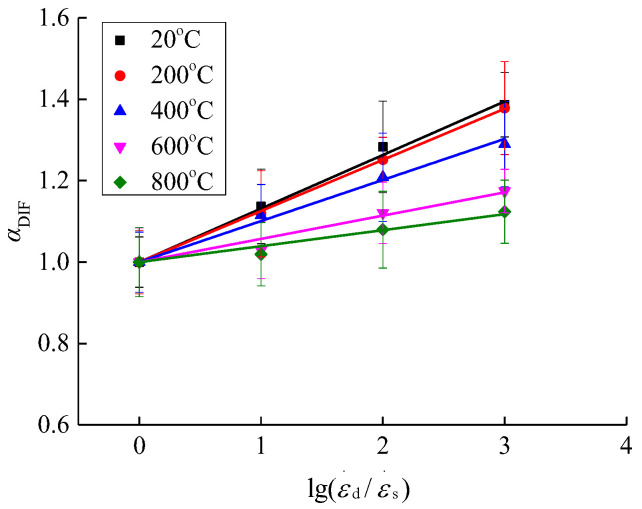
Relationship between strain rate and concrete dynamic increase coefficient.

The compressive stress was extracted from the compressive stress-strain curves of concrete under different temperatures and strain rates in this paper, and the changing trend of the compressive stress affected by strain rate after rapid cooling at different temperatures was analyzed, as shown in Figure 10 and Figure 11.

According to Figure 10, when the temperature is 20 °C, the compressive stress is increased from 35.69 MPa at the static strain rate of 10^−5^/s to 49.50 MPa at the strain rate of 10^−2^/s, indicating an increase by 38.69% due to the strain rate. When the temperature is 200 °C, the compressive stress is increased from 33.58 MPa at the static strain rate of 10^−5^/s to 46.29 MPa at the strain rate of 10^−2^/s, indicating an increase by 37.85% due to the strain rate. When the temperature is 400 °C, the compressive stress is increased from 27.96 MPa at the static strain rate of 10^−5^/s to 36.07 MPa at the strain rate of 10^−2^/s, indicating an increase by 29.01% due to the strain rate. When the temperature is 600 °C, the compressive stress is increased from 17.94 MPa at the static strain rate of 10^−5^/s to 21.07 MPa at the strain rate of 10^−2^/s, indicating an increase by 17.45% due to the strain rate. When the temperature is 800 °C, the compressive stress is increased from 9.77 MPa at the static strain rate of 10^−5^/s to 10.98 MPa at the strain rate of 10^−2^/s, indicating an increase by 7.78% due to the strain rate. In the relevant literature [30,31], the mechanical properties of ordinary concrete under low to medium strain rates (10^−5^/s~10^−2^/s) under compressive stress have been investigated. Compared with static conditions, the compressive stress is generally increased by 30% to 40% by the strain rate. In this paper, the increase in compressive stress affected by the strain rate under normal temperature conditions is consistent with the references above, but there are some differences in the increasing range among different reports, which are mainly related to the randomness and discreteness of concrete materials. Bi et al. [19] used Hopkinson bars to conduct a dynamic stress study on concrete under natural cooling and rapid cooling under high strain rate conditions, and reported that the increase in compressive stresses under rapid cooling was higher than that under natural cooling. The literature [32] reviewed the coupling effect of high temperature and high strain rate on the dynamic properties of concrete under natural cooling, and the results showed that when the temperature exceeded 400 °C, the compressive stress was increased by the strain rate and decreased significantly and gradually. Based on the analysis above, it can be seen that, after the same temperature treatment, the compressive stress value is gradually increased as the strain rate increases. The increase in temperature makes the concrete compressive stress affected by the strain rate increase significantly and gradually decrease. When the temperature exceeds 400 °C, the compressive stress is significantly reduced by the strain rate. Temperature and cooling methods have obvious effects on the compressive performance of concrete. Due to the large temperature gradient generated by the rapid cooling method and higher temperature, the compressive stress of concrete is affected by the strain rate. Ultimately, the increase in the compressive stress of concrete affected by the strain rate is relatively insignificant compared to the natural cooling method and the normal temperature method.

The effect of strain rate on the dynamic response of concrete under compressive stress is generally expressed quantitatively by using the dynamic increase factor α_DIF_, which is expressed in the form shown in Equation (1) [25,26].

(1) $$\alpha_{\mathrm{DIF}}=\sigma_{d}/\sigma_{s}$$

where *σ*_d_ is the compressive stress of concrete under different dynamic strain rates after high temperature action, unit: MPa; *σ*_s_ is the compressive stress of concrete under different static strain rate conditions after high temperature action, unit: MPa.

According to Figure 10 and combined with Equation (1), the dynamic increase factor of concrete after different high temperature effects was obtained, as shown in Figure 11. The relationship between the compressive stress and strain rate of concrete under low and medium strain rates is generally expressed in the form of Equation (2) to quantitatively describe the relationship between the dynamic increase factor and strain rate [20,25].

(2) $$\alpha_{\mathrm{DIF}}=a+b\mathrm{lg}({\dot{\varepsilon }}_{d}/{\dot{\varepsilon }}_{s})$$

where the parameter *a* represents the compressive stress dynamic increase coefficient under the static strain rate (according to actual analysis, *a* generally takes one); the parameter *b* represents the increase range of compressive stress affected by the strain rate. Based on the dynamic increase factor of concrete after different high temperature and rapid cooling treatments in this paper, mathematical regression analysis was performed by applying Equation (2), and the equations for the relationship between the dynamic increase factor and strain rate of concrete after rapid cooling at different high temperatures were obtained as shown in Figure 11 and Equations (3)–(7).

(3) $$T=20\;{}^{\circ}C\;\;\;\;\alpha_{\mathrm{DIF}}=1+0.1316\mathrm{lg}({\dot{\varepsilon }}_{d}/{\dot{\varepsilon }}_{s})$$

(4) $$T=200\;{}^{\circ}C\;\;\;\alpha_{\mathrm{DIF}}=1+0.1256\mathrm{lg}({\dot{\varepsilon }}_{d}/{\dot{\varepsilon }}_{s})$$

(5) $$T=400\;{}^{\circ}C\;\;\;\alpha_{\mathrm{DIF}}=1+0.1010\mathrm{lg}({\dot{\varepsilon }}_{d}/{\dot{\varepsilon }}_{s})$$

(6) $$T=600\;{}^{\circ}C\;\;\;\alpha_{\mathrm{DIF}}=1+0.0571\mathrm{lg}({\dot{\varepsilon }}_{d}/{\dot{\varepsilon }}_{s})$$

(7) $$T=800\;{}^{\circ}C\;\;\;\alpha_{\mathrm{DIF}}=1+0.0393\mathrm{lg}({\dot{\varepsilon }}_{d}/{\dot{\varepsilon }}_{s})$$

**Figure 12:**
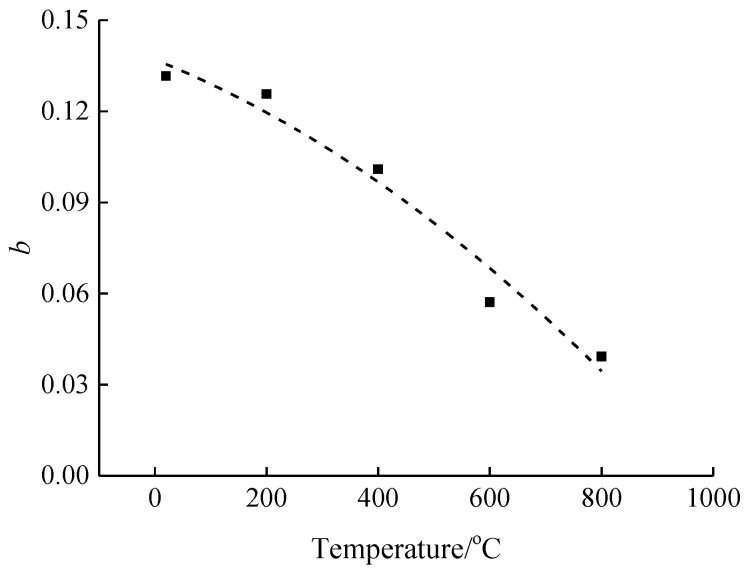
The relationship between temperature and parameter *b*.

According to Figure 11, Equation (2) proposed in this paper can well describe the relationship between the compressive stress dynamic increase factor and strain rate of concrete after different high temperature and rapid cooling treatments. The parameter *b* tends to decrease gradually with the rise of temperature, thus indicating from a quantitative point of view that the concrete dynamic increase factor is gradually reduced by the strain rate as the temperature increases. This is consistent with the qualitative conclusion. In order to illustrate the coupling effect of strain rate and temperature on the dynamic increase factor, the parameter *b* was expressed quantitatively with temperature *T* and the expression form as shown in Figure 12 was obtained.

According to the conclusion of the qualitative analysis of temperature and the dynamic increase coefficient of concrete compressive stress, when the temperature is lower than 400 °C, as the temperature increases, the increase of the compressive stress of concrete at different temperatures is relatively small and decreases due to the influence of the strain rate. When the temperature exceeds 400 °C, with the rise of temperature, the change in compressive stress affected by the strain rate is increased significantly under different temperatures. Based on the relationship between the temperature and parameter *b* in Figure 12, the expression form as shown in Equation (8) was proposed.

(8) $$b=0.1370-7.34\times 10^{-5}\times T-6.86\times 10^{-8}\times T^{2}$$

Substituting Equation (8) into Equation (2) gives the relationship equation of the coupling effect of temperature and strain rate on the dynamic increase factor of compressive stress, as shown in Equation (9).

(9) $$\alpha_{\mathrm{DIF}}=1+(0.1370-7.34\times 10^{-5}\times T-6.86\times 10^{-8}\times T^{2})\times \mathrm{lg}({\dot{\varepsilon }}_{d}/{\dot{\varepsilon }}_{s})$$

### 3.5. Deformation Analysis

#### 3.5.1. Elastic Modulus

The elastic modulus *E* is one of the important characteristic values in the research on the mechanical properties of concrete. In this paper, the elastic modulus of concrete was calculated according to the compressive stress-strain curves under different working conditions, as shown in Equation (10).

(10) $$E=\frac{\sigma_{0.5}}{\varepsilon_{0.5}}$$

where, *σ*_0.5_ refers to 50% of the concrete peak stress, unit: MPa; *ε*_0.5_ is the strain value corresponding to 50% of the concrete peak stress.

**Figure 13:**
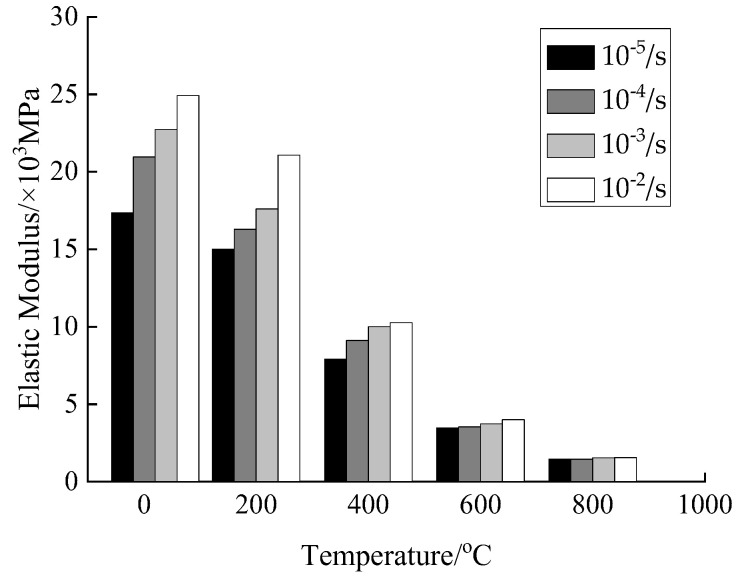
Elastic modulus of concrete.

**Figure 14:**
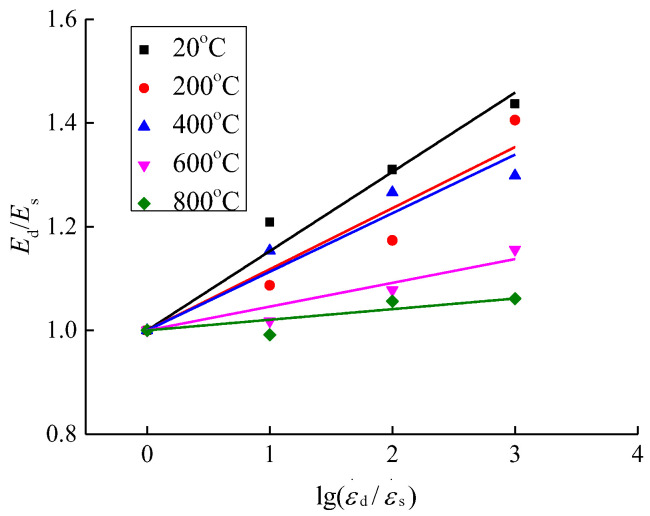
Change factor of elastic modulus.

In order to examine the relationship between the elastic modulus of concrete affected by the strain rate after different high temperatures and rapid cooling treatments, the same method as the dynamic increase factor of compressive stress was used in this paper to analyze the elastic modulus, that is, to define the change factor of the elastic modulus *E*_d_/*E*_s_ (*E*_d_ is the elastic modulus under the dynamic strain rate and *E*_s_ is the elastic modulus under the static strain rate). Thus, the expressions as shown in Figure 13 and Figure 14 were obtained.

According to Figure 13 and Figure 14, when the temperature is 20 °C, the elastic modulus of concrete is increased from 17.34 × 10^3^ MPa at the strain rate of 10^−5^/s to 24.92 × 10^3^ MPa at the strain rate of 10^−2^/s, indicating an increase by 43.67% affected by the strain rate. When the temperature is 200 °C, the elastic modulus is increased from 15.00 × 10^3^ MPa at the strain rate of 10^−5^/s to 21.08 × 10^3^ MPa at the strain rate of 10^−2^/s, indicating an increase by 40.53% affected by the strain rate. When the temperature is 400 °C, the elastic modulus is increased from 7.90 × 10^3^ MPa at the strain rate of 10^−5^/s to 10.26 × 10^3^ MPa at the strain rate of 10^−2^/s, indicating an increase by 29.84% affected by the strain rate. When the temperature is 600 °C, the elastic modulus is increased from 3.47 × 10^3^ MPa at the strain rate of 10^−5^/s to 4.01 × 10^3^ MPa at the strain rate of 10^−2^/s, indicating an increase by 15.60% affected by the strain rate. When the temperature is 800 °C, the elastic modulus is increased from 1.46 × 10^3^ MPa at the strain rate of 10^−5^/s to 1.55 × 10^3^ MPa at the strain rate of 10^−2^/s, indicating an increase by 6.1% affected by the strain rate. In the relevant literature [20,25,26], research has been carried out on the elastic modulus of ordinary concrete at low and medium strain rates (10^−5^/s~10^−2^/s). Compared with static conditions, the elastic modulus of concrete is generally increased by 25–45% affected by the strain rate. In this paper, the changing range of the elastic modulus affected by the strain rate under normal temperature conditions is consistent with this interval. As the concrete deformation parameters are influenced by the material randomness, it leads to a more discrete variation of the elastic modulus affected by the strain rate.

Based on the above analysis, the elastic modulus of concrete is gradually reduced with the rise of temperature under the same strain rate, and is gradually increased with the increase of strain rate under the same temperature. The increase in temperature gradually weakens the effect of strain rate on the elastic modulus of concrete.

Based on the same model equations as the effect of strain rate on the dynamic increase factor of compressive stress, mathematical regression analysis was performed on the elastic modulus of concrete under different working conditions and the expression forms as shown in Figure 14 and Equations (11)~(15) were obtained.

(11) T=20 °C   EdEs=1+0.1528lg(ε.d/ε.s)

(12) T=200 °C   EdEs=1+0.1178lg(ε.d/ε.s)

(13) T=400 °C   EdEs=1+0.1130lg(ε.d/ε.s)

(14) T=600 °C   EdEs=1+0.0458lg(ε.d/ε.s)

(15) T=800 °C   EdEs=1+0.0205lg(ε.d/ε.s)

From Figure 14 and Equations (11)~(15), it can be seen that the model equation proposed in this paper has great applicability for quantitatively expressing the relationship between the strain rate and the elastic modulus change factor. As the temperature increases, the slope parameters of Equations (11)~(15) are gradually decreased. From a qualitative point of view, it shows that the increase in elastic modulus affected by the strain rate is gradually reduced as the temperature increases.

#### 3.5.2. Peak Strain

**Figure 15:**
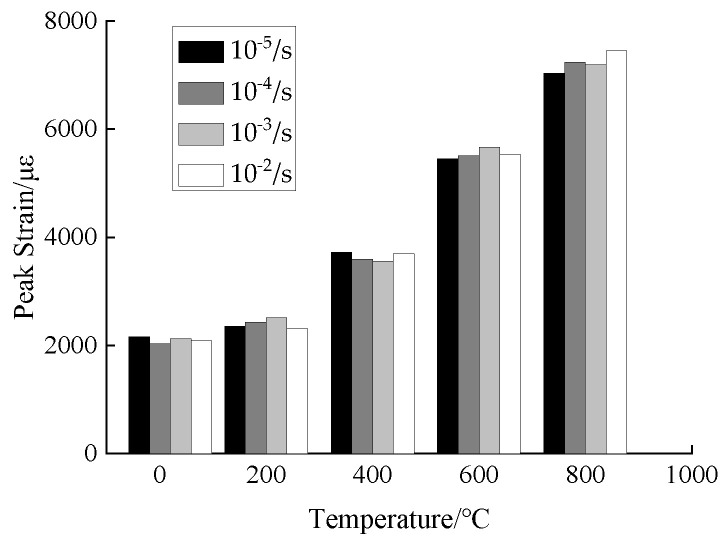
Peak strain of concrete.

**Figure 16:**
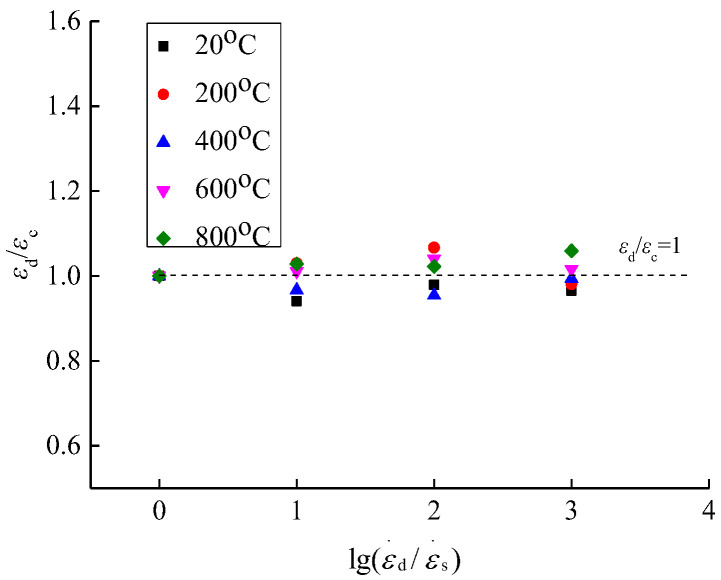
Change factor of peak strain.

The peak strain was extracted from the compressive stress-strain curves of concrete under different temperatures and strain rates, and the effects of temperature and strain rate on the peak strain of concrete were analyzed, as shown in Figure 15 and Figure 16.

From the analysis of Figure 15 and Figure 16, when the temperature is 20 °C and the strain rate is in the range of 10^−5^/*s*~10^−2^/*s*, the compressive peak strain of concrete is in the range of 2037~2166 μ*ε*, indicating a changing range of −2.08−5.96%. When the temperature is 200 °C, the compressive peak strain is in the range of 2312~2514 μ*ε* under different strain rates, indicating a changing range of −1.91~6.66%. When the temperature is 400 °C, the compressive peak strain is in the range of 3556~3726 μ*ε* under different strain rates, indicating a changing range of −0.64−4.56%. When the temperature is 600 °C, the compressive peak strain is in the range of 5549~5668 μ*ε* under different strain rates, indicating a changing range of 1.12%~4.02%. When the temperature is 800 °C, the compressive peak strain is in the range of 7037~7454 μ*ε* under different strain rates, indicating a changing range of 2.25~5.93%. From the overall trend analysis, as the temperature increases, the compressive peak strain of concrete is gradually increased and the plastic deformation capacity of concrete is gradually improved.

After the same temperature action, the peak strain of concrete shows a discrete changing trend with the increase of strain rate. In the literature about peak strain affected by low and medium strain rates, the conclusions present a large variability as follows: (1) Under low and medium strain rates, the peak strain of concrete is gradually increased as the strain rate increases; (2) as the strain rate increases, the peak strain of concrete is gradually reduced; (3) as the strain rate increases, the peak strain of concrete shows a discrete changing trend. This is mainly due to the coupling effect of the randomness and correlation of discrete ratios for concrete. Under high strain rate conditions, the compressive peak strain of concrete is significantly higher than that under static conditions, and the compressive peak strain is gradually increased with the increase of strain rate [20,25,26].

## 4. Microscopic Mechanism

### 4.1. SEM Technology

**Figure 17:**
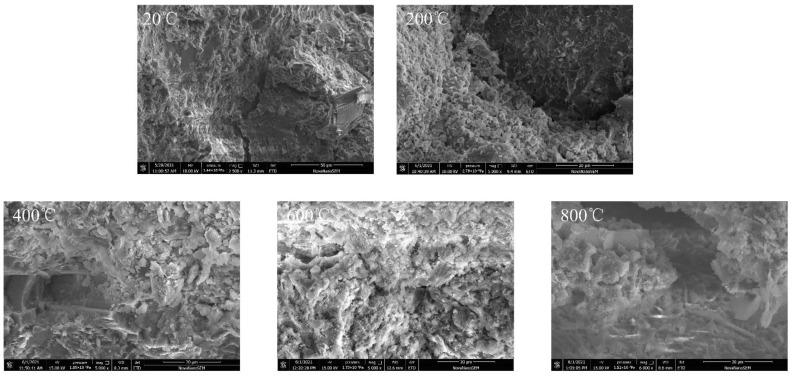
SEM microscopic morphology of concrete after high temperature and rapid cooling.

By using scanning electron microscopy (SEM) technology, microscopic tests were carried out on the test specimens treated at five different temperatures in this paper, so as to analyze the changes in the microscopic composition of concrete after high temperature and rapid cooling, as shown in Figure 17.

According to Figure 17, when the temperature is 20 °C, a large amount of flocculent calcium silicate hydrate can be observed from the scanning electron microscope photos; a small amount of flake calcium hydroxide deposits on the calcium silicate hydrate and some ettringite can also be observed; the scan results show a denser microstructure. When the temperature is 200 °C, the microstructure shows a certain number of cracks, and there is a large amount of hydride filling during this period. When the temperature is 400 °C, from SEM photos we can see the cracks in the specimen significantly increase; at the same time, a large number of new short needles are produced and distributed in the fissures, which are mainly the new calcium silicate hydrates formed by the combining action of temperature and moisture. When the temperature is 600 °C, a large number of floccules can be observed from the SEM images. The floccules are mainly secondary hydrates after rapid cooling, and it is difficult to observe the flakes. When the temperature is 800 °C, the internal structure of the test specimen is gradually loosened by high temperature. After rapid cooling, moisture can quickly enter into the specimen, which triggers further hydration reactions. From the microstructure, more fine needles as well as radial hydrides can be observed, and the loose structure leads to the formation of obvious gaps.

### 4.2. CT Scanning

**Figure 18:**
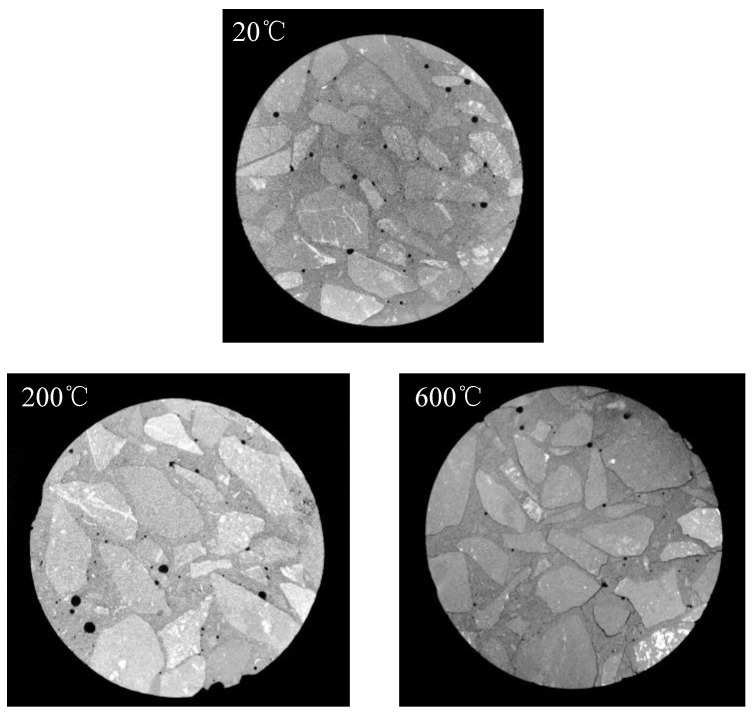
Microscopic morphology of concrete after high temperature and rapid cooling by using CT scanning.

By applying CT scanning technology, non-destructive scanning observation was performed on the test specimens after different high temperature and rapid cooling treatments, and the concrete sections at 20 °C, 200 °C and 600 °C were obtained as shown in Figure 18.

According to the analysis in Figure 18, when the temperature is 20 °C, the CT scanning section shows relatively smaller pores, which are mainly formed by the evaporation of free water inside the test specimen during the concrete curing process. The pores in this part, as well as the interfacial mechanical properties of the mortar and coarse aggregate, lead to the randomness characteristics of the concrete. When the temperature is 200 °C, the CT scanning section is basically the same as the section formed at 20 °C, but the pore size is larger than that at 20 °C. This is mainly due to the further evaporation of free water after the curing process following the action of 200 °C temperature. When the temperature is 600 °C, the CT scanning section becomes significantly different. The interface between the mortar and coarse aggregate in the concrete is obviously peeled off, and there is a clear gap between the two media. It is mainly due to the inconsistency of deformation between the mortar and coarse aggregate after being subjected to high temperature, resulting in poor deformation and finally formation of a gap.

Concrete is prone to forming a uniform stress state under the action of low strain rate compression, so that the stress tends to develop through the weak areas of the specimen. The concrete failure generally occurs at the interface between the mortar and coarse aggregate. When the strain rate is high, the compressive stress of concrete cannot form a uniform stress state in a short period of time, and will spread through the axial stress rapidly, resulting in the destruction of parts of coarse aggregates. Eventually, the compressive stress of concrete is increased under the action of high strain rates. When concrete is subjected to high temperature, a gap will be formed between the mortar and coarse aggregate. Under the action of low strain rates, the concrete stress can easily pass through the interface between the mortar and coarse aggregate, causing parts of course aggregates to peel off. When the strain rate is high, the concrete stress state will propagate to the mortar and coarse aggregate interface to form stress propagation paths in the interface weak zone and in the mortar fractures. The final damage pattern is basically similar to that under low strain rates, which reflects the concrete mechanical properties from a macroscopic perspective. It leads to a gradual weakening of the concrete strain rate effect under compressive stress as the temperature increases.

## 5. Constitutive Model under High Temperature Dynamics

For materials such as plexiglass, nylon and epoxy resin, the relevant literature has proposed to use the ZWT model to describe their constitutive model [33]. The ZWT model has good applicability in establishing the concrete dynamic constitutive model, which can better describe the effect of strain rate on the stress-strain curve of concrete [34]. The expression form of ZWT is shown in Equation (16).

(16) $$\sigma =E_{0}\varepsilon +\alpha \varepsilon^{2}+\beta \varepsilon^{3}+E_{{}^{1}}\int_{0}^{\tau }\dot{\varepsilon }e^{\left(-\frac{t-\tau }{\theta_{1}}\right)}d\tau +E_{2}\int_{0}^{\tau }\dot{\varepsilon }e^{\left(-\frac{t-\tau }{\theta_{2}}\right)}d\tau$$

where *σ* is the stress; *ε* is the strain; *έ* is the strain rate; *E*_0_ is the Young’s modulus in the elastic state; *α* and *β* are the material constants; *E*_1_ and *θ*_1_ are the elastic modulus of viscoelastic response at low strain rates and the corresponding relaxation time; *E*_2_ and *θ*_2_ are elastic modulus of viscoelastic response at high strain rates and the corresponding relaxation time; *τ* is the time constant.

In this paper, the ZWT model was applied to build the dynamic constitutive model of concrete after rapid cooling from different high temperatures. The relaxation time *θ*_2_ was taken to be in the range of 10^−5^/s to 10^−2^/s, and the time of the dynamic compression test would last much longer than *θ*_2_. After the start of the test, the viscoelastic response term at the high strain rate of *θ*_2_ would relax immediately. Because the strain rate is too small (10^−5^/s~10^−2^/s), the integral term of viscoelastic response under high strain rates can be ignored, and Equation (16) can be simplified to Equation (17).

(17) $$\sigma =E_{0}\varepsilon +\alpha \varepsilon^{2}+\beta \varepsilon^{3}+E_{{}^{1}}\dot{\varepsilon }\theta_{1}[1-e^{\left(-\frac{\varepsilon }{\dot{\varepsilon }\theta_{1}}\right)}]$$

The concrete compression test under the strain rate of 1.0 × 10^−5^/s is considered as a quasi-static test, and the model parameters are fitted using Matlab. Under the same temperature, the stress corresponding to the same strain on the stress-strain curve at the strain rates of 1.0 × 10^−4^/s, 1.0 × 10^−3^/s and 1.0 × 10^−2^/s was extracted. By eliminating the material pure elastic response term *E*_0_*ε* + *αε*_2_ + *βε*_3_ in Equation (17), Equation (18) was obtained. Fitting parameters *E*_1_, *θ*_1_, then substituting *E*_1_, *θ*_1_ into Equation (17) and using the stress-strain curve in the quasi-static test to fit the parameters *E*_0_, *α* and *β*, the expression form of Equation (18) can be obtained.

(18) $$\sigma_{2}-\sigma_{1}=E_{{}^{1}}\dot{\varepsilon_{2}}\theta_{1}[1-e^{\left(-\frac{\varepsilon }{\dot{\varepsilon_{2}}\theta_{1}}\right)}]-E_{{}^{1}}\dot{\varepsilon }\theta_{1}[1-e^{\left(-\frac{\varepsilon }{\dot{\varepsilon_{1}}\theta_{1}}\right)}]$$

where *σ*_2_ and *έ*_2_ are the dynamic strain rate and stress under the static dynamic strain rate, respectively; *σ*_1_ and *έ*_1_ are the static strain rate and stress under the static strain rate, respectively.

During concrete loading under compression, concrete is in the elastic and elastoplastic phase before reaching the peak compressive stress. After the peak stress is reached, the concrete will be in the decreasing phase. For concrete under compression, it is difficult to obtain the exact decreasing phase due to the unloading stiffness of loading equipment. Based on the ZWT model, a dynamic principal structure model of concrete has been proposed, which is generally considered to be able to simulate and calculate the stress-strain curve before the peak stress point of concrete under compression. Combining the various reasons above, this paper mainly calculated the phase before the peak stress point of the stress-strain curve under compression based on the ZWT model.

**Figure 19:**
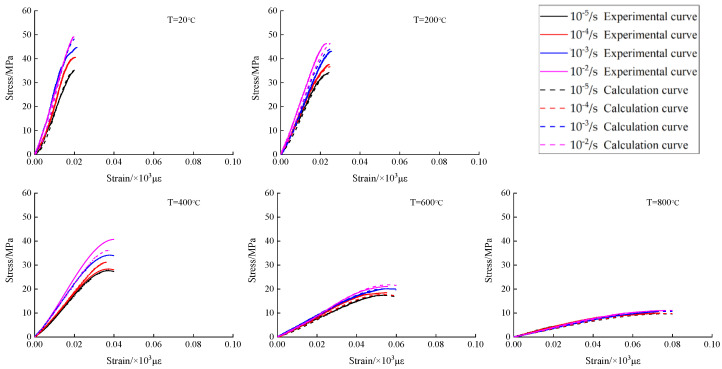
Comparison of test curves and calculated curves under dynamic strain rates of concrete after high temperature and rapid cooling.

The concrete model parameters *E*_0_, *E*_1_, *α*, *β* and *θ*_1_ at different temperatures were obtained by mathematical regression. By substituting these parameter values into Equation (17), the concrete dynamic constitutive model was obtained. Figure 19 shows a comparison of the stress-strain curves and the model prediction curves at different temperatures. As can be seen from Figure 19, the model prediction curves are in good agreement with the experimental curves, indicating that the model can better describe the mechanical behavior of concrete at different temperatures under low strain rates.

## 6. Conclusions

Based on different high temperature rapid cooling treatments (normal temperature/20 °C, 200 °C, 400 °C, 600 °C and 800 °C) and low strain rates (10^−5^/s, 10^−4^/s, 10^−3^/s and 10^−2^/s), an experimental study on the compressive properties of concrete was conducted in this paper. Combining with the results of microscopic experiments and analysis of the coupling effect of high temperature and strain rate on the mechanical properties of concrete, the following conclusions were obtained:

(1) When the temperature is low, the damage morphology of concrete changes from diagonal cracks under the static strain rate to longitudinally distributed cracks under the dynamic strain rate. A small amount of coarse aggregate is damaged under high strain rate working conditions. When the temperature is high, the damage morphologies under the static dynamic strain rate all show an oblique pattern. The final damage pattern of concrete under the action of different strain rates is basically the same, which is the interfacial damage between the mortar and coarse aggregate accompanied by coarse aggregate spalling.
(2) Under static loading strain rate conditions, as the temperature increases, the compressive stress of concrete is gradually reduced and the plastic deformation capacity is gradually increased. When the temperature exceeds 400 °C, the compressive stress decreases quickly, and the surface of the specimen gradually shows an increasing number of obvious cracks. Compared with natural cooling, the compressive stress attenuation under rapid cooling is relatively large, which is directly related to the fact that the temperature gradient can be more easily formed after rapid cooling.
(3) Under the same temperature, with the increase of strain rate, the compressive stress and elastic modulus of concrete are gradually increased, but the peak strain shows a discrete changing trend affected by the strain rate. When the temperature is 20 °C, 200 °C, 400 °C, 600 °C and 800 °C, the compressive stress affected by the strain rate is increased by 38.69%, 37.85%, 29.01%, 17.45% and 7.78%, respectively. The increase of temperature leads to a gradually decreasing trend of the compressive stress affected by the strain rate. The main reason is that cracks are formed in the concrete after the action of high temperature, and the cracks will directly affect the stress path propagation of the concrete during the stress process.
(4) SEM and CT scanning technologies were used to analyze the test specimens after different temperatures. Combined with the conclusions of macro-mechanical performance analysis, the coupling effect of high temperature and strain rate on the mechanical properties of concrete was revealed. At the same time, based on the ZWT model, a dynamic constitutive model of concrete after different high temperature and rapid cooling treatments was proposed, which has been demonstrated to have good applicability.

## Figures and Tables

**Table 1:** Mix proportion of C40 strength grade concrete.

Strength Grade	Raw Materials Used in the Concrete Mixture Per Unit Volume (kg/m^3^)			
C40	Cement	Water	Fine aggregate	Coarse aggregate
	450	202	670	1095

## Data Availability

Not applicable.
